# Hypersensitivity of Dentine and Its Treatment

**Published:** 1897-01

**Authors:** Henry H. Burchard

**Affiliations:** Philadelphia


### International Dental Journal.

Vol. XVIII. January, 1897. No. 1.

### Original Communications.[1]

### HYPERSENSITIVITY OF DENTINE AND ITS TREATMENT.[2]

BY HENRY H. BURCHARD, M.D., D.D.S., PHILADELPHIA.

The study of disordered or exalted function of any part is logically preceded by a study of the normal function and relations of the part.

Investigations as to the sources of sensation itself, and a parallel study of the mechanism of sensation, divide the nervous system into three parts, so far as the study of anæsthesia is concerned. The first factor involved is a receptive apparatus, or that through which sensation is primarily received; the second is perceptive, or the anatomical region in which there arises consciousness of sensation; the third considers the pathway or the mode of transmission from the first to the second.

The first, the receptive apparatus, includes the several nerve-endings of sensory nerves; the second, the path of transmission, includes the nerve-fibres and nerves which begin in these endings and pass into the posterior columns of the spinal cord, if spinal nerves, or through corresponding paths if they be cranial nerves, and have their apparent physiological endings in the perceptive

[1] The editor and publishers are not responsible for the views of authors of papers published in this department, nor for any claim to novelty, or otherwise, that may be made by them. No papers will be received for this department that have appeared in any other journal published in the country.

[2] Read before the Academy of Stomatology, Philadelphia, November 24, 1896.

areas of the brain,—areas in the right and left hippocampal regions. Sensation may be defined as a stimulus received by a receptive nerve-ending, transmitted by the path named to this area when it becomes translated into conscious sensation.

Stimuli, productive of what is termed normal sensation, may be regarded as a definite quantity; an increase in the degree of stimulation is productive of disordered sensation, and if the degree be high enough give rise to what is termed pain. It is not absolutely necessary that the stimuli producing pain be received by the terminals, whose function is the reception of such stimuli. The clinical records of medicine and of dentistry afford numerous instances in which the stimulus causing pain has been received at some portion of the transmitting apparatus. This factor needs no further comment at this point, as its study is the study of surgical neuralgia; suffice to say that when the source of irritation is in some portion of the transmitting apparatus, the perceptive centre may refer the sensation to any portion of the distribution of the nerve affected, which states that the farther from the proper receptive apparatus the cause of irritation is, the more difficult it is to locate the cause.

As pointed out and elucidated by Dr. Black ("American System of Dentistry," vol. i.), organs which have no tactile receptive organs proper have, as a rule, stimuli received by their nerve terminals referred to other parts; in a word, they are distinguished by reflected sensations. The dental pulp is such an organ, and it is in the experience of every dentist that irritation beginning in exposure of the dentine periphery of a tooth and ending in degenerations of the pulp itself are not precisely located, but are referred to other parts, usually to some portion of the fifth cranial nerve.

Although the mutual interactions which physiologists and pathologists describe as occurring between disordered sensation and nutrition are of direct clinical interest, their discussion is beyond the province of the present essay. Nor is the discussion of relative perception in order; hypersensitivity of dentine is defined as such a condition of æsthesia of dentine as precludes the cutting away of such an amount of dentine as required for the perfect preparation of a cavity for filling. What difference in the irritability there may exist in the dentine of different individuals, or whether the differences are psychical, will form no factor in our studies. We simply accept that the objective evidences of pain evinced in subjective signs may be divided into groups,—*i.e.*, there is evidence of wide differences as the sensitivity of the dentine.

Reverting to the statement that there are three factors concerned in sensation or æsthesia, it is a corollary that the same three are concerned in the induction of anæsthesia.

The first of these that has the widest range of effect is, of course, the perception, for, although pain, or rather the conditions of pain, may exist objectively, they have no existence subjectively until the perceptive centre takes cognizance of them, and it follows, therefore, that if the function of perception be abolished no pain can be felt, no matter what irritants may be applied to the receptive or transmissive apparatus. The first great means, therefore, of destroying or preventing pain is holding in abeyance the function of the perceptive centre. Agents which have the power to accomplish this end are comprised in the classes of general anæsthetics and general anodynes.

Of general anæsthetics, the inhalation of chloroform, of ether, and seldom of nitrous oxide have been employed to lessen the perception of the irritation caused by the cutting of hypersensitive dentine. Chloroform is, of course, excluded from dental practice as an anæsthetic, even when used in small amounts, owing to its attendant dangers.

Dr. Bonwill has stated to the writer that as early as 1855 he has practised chloroform inhalation as a means of procuring dentinal insensitivity.

The vapor of ether has been employed with marked success. It should be noted in this connection that the apparatus devised by Dr. Fillebrown, shown at one of our meetings last year, materially broadens the field of the usefulness of ether in this immediate direction.

Of general anodynes two have been used which act upon the perceptive centre.

The first of these is morphine, which, for the specific purpose under discussion, is combined with atropine, which lessens its soporific effect without diminishing the anodyne property; the amounts of the drug used are,—

℞ Morphiæ Sulph., gr. $\frac{1}{8}$;

Atropiæ Sulph., gr. $\frac{1}{150}$. M.

Sig.—Half-hour before operating.

The administration of this prescription has materially lessened the pain incident to the cutting of hypersensitive dentine. The next of the general anodynes are the coal-tar derivatives. Acetanilide is taken as the basis of these; the general effects of the proprietary preparation called antipyrin, and also of phenacetin, and acetanilin are almost alike. The basal drug is combined with others which modify its effects, preserving, however, its two great properties, pain and temperature reducing. Perhaps the best of these preparations is ammanol, which, containing ammonium carbonate, removes any unpleasant symptoms referable to the heart.

The dose, ten grains, administered one-half hour before operating, has been found to reduce the perception of dental pain in some cases, the drug acting as morphia upon the perceptive centre.

Under this heading the induction of the hypnotic state to prevent pain perception belongs, as hypnotism is essentially the directing of psychic perception, so that the functions of the lower centres are placed under domination. Whatever conception and classification the psychologist may have as to hypnotism, this is its function in our immediate practice. I pass over this subject without comment, owing to a feeling of indecision as to the ethical side of it, not owing to any doubt as to its effectiveness.

The rapid respiration method of Bonwill should also be included under the head of general anæsthetics.

The next class of agents are those acting upon the apparatus of transmission. Two drugs may be selected for example in this connection, the first, the coal-tar derivatives, just discussed, which appear to interfere with the transmission as well as the perception of pain; the second, cocaine.

In order to secure this effect with cocaine it is necessary that it be brought into contact with the apparatus of transmission. This end may be accomplished in two ways, the first the direct injection of a solution of cocaine near the apparatus of transmission or its introduction to the nerve trunk by means of the cataphoric current.

The first of these means has been tried with a varying degree of success, the second is still open for experiment. It cannot be too strongly emphasized that in the injection of cocaine for operations about the mouth, two precautions should always be taken. The first, the careful sterilization of the field of operation, and of the syringe employed; the second, the use of the minimum physiological dose of the alkaloid. The first end is accomplished by first scrubbing the teeth and gums with a soft brush and an antiseptic soap, following this by a spray of hydrogen peroxide or a ten-per-cent. solution of meditrina, the syringe to be soaked well in a five-per-cent. solution of carbolic acid, or in listerine.

The amount of cocaine employed should not be greater than one-eighth grain dissolved in—

℞. Aquæ, ♏xl;

Listerine, ♏xx. M.

It may be remarked that surgical accidents are far from uncommon, following cocaine injections about the jaws. Dr. Cryer has, I believe, some interesting data in this connection.

The third method of destroying or preventing pain is that usually employed by the dental operator for the obtunding of hypersensitive dentine,—*i.e.*, abolishing the function of the receptive apparatus.

We are all perfectly familiar with the fact that any and every agent which has been shown to have the power of lessening pain through local application or of temporarily benumbing nerve terminals has been used for the purpose under discussion. The most prominent of the agents which have been employed may be conveniently grouped under heads, beginning with the mildest agents.

The first means of lessening the irritability of nerve terminals is, of course, the removal of the substance which irritates them. It has long been held, as many of us are aware, that the direct cause of hypersensitivity of dentine is an acid condition, the phrase being applied in a somewhat indefinite manner. In accordance with this belief it has been and is a common practice to use alkaline mouth-washes and apply mild alkalies to the hypersensitive parts, and in many cases marked relief followed. The agents most commonly employed are a mouth-wash of lime-water, and precipitated chalk,—calcium carbonate. In view of our present knowledge of the pathology of caries and of oral bacteriology, it is a perfectly reasonable hypothesis that the source of irritation in early caries is the irritation of the terminal filaments of the dentinal processes by lactic acid; the use of an alkali by neutralizing the acid removes the source of irritation. The lactate of calcium is formed from the chalk instead of from the calcium of the teeth.

The other agents which have been used as obtundents may be included in four groups, according to their mode of action. First, cold; second, heat; third, specific obtundents, and fourth, agents which destroyed the receptive apparatus.

*First, Cold.*—It is a well-recognized fact in general and special practice that the lowering of the temperature of a part is followed by a diminished functional activity of the part. It has long been known that the literal freezing of a part was followed by an abolition of sensation in the part frozen.

This principle has been made use of in dentistry at earlier periods for the extraction of teeth, and within the past ten years for the obtunding of hypersensitive dentine; as this variety of dentine, or, more specifically, the dental pulp is peculiarly intolerant of sudden changes of temperature; an apparatus is required which will lessen the temperature by almost imperceptible gradations.

The purpose is accomplished by directing a spray of one of the highly volatile hydrocarbons against the exposed dentine. Rhigolene, ether, and chloroform, which had former employment in this connection, have been superseded by more volatile agents,—those having a less molecular weight,—ethyl and methyl chloride and pental. These agents, although general anæsthetics, are too volatile and evanescent to be generally serviceable in that direction. The bottle or vial in which the liquid is contained constitutes the apparatus required after isolation of the tooth by rubber dam. In some cases a degree of insensibility may be produced which will permit the painless exposure or even extirpation of the pulp.

The rapid vaporization of liquid nitrous oxide may be utilized in a similar manner.

*Second, Heat.*—Heat, as dental practice testifies, is an obtundent agent, acting not as cold or as a specific analgesic, but in virtue of its drying property, which states, of course, that dry heat is to be used.

It is applied as blasts of dry, hot air by means of appropriate apparatus after the tooth has been isolated. Dr. Register introduced some years ago a method and apparatus with which many of us are familiar, by means of which a blast of hot, dry air was forcibly driven against the walls of hypersensitive dentine, and produced a marked obtundent effect. Members of the State Dental Society will recall an able and instructive paper written by Dr. C. V. Kratzer upon this subject. It is very probable that the contents of the dentinal tubuli are shrunken through being deprived of a portion of their water, and thus are prevented from performing their normal function, the reception of stimuli.

The next means of applying heat as an obtundent might, perhaps, be more properly classed under those agents which destroy the receptive mechanism. It is by applications of hot burnishers, the method being followed only in those cases of hypersensitivity of the dentine upon the periphery, such as the abraded masticating surfaces of the teeth. The dental cautery has also been used for the same purpose; the loop of platinum, made almost white hot, is passed over the hypersensitive spots.

*Third.*—The next class of agents or measures used are those which chemically destroy the receptive apparatus. Under this head are included the powerful cauterants, such as zinc chloride, silver nitrate, the mineral acids, and strong alkalies, carbolic acid, creosote, and allied substances, and chromic acid. Some of these agents, such as chromic acid, zinc chloride, aqua ammoniæ fortior, sodium carbonate and hydrate, are notably hygroscopic, abstracting water from bodies with which they are brought in contact; most of those named abstract water as water from the tissues; still others of the group, notably sulphuric acid, has such an affinity for water that it extracts the elements of water from even solid carbohydrates,—as, for example, when brought in contact with sugar, a stable chemical compound, where the water does not occupy the position of water of crystallization, but is an integral part of the compound itself; sulphuric acid extracts the elements of water and leaves the carbon,—

$$C_6H_{12}O_6 + H_2SO_4 = H_2SO_4 + 6H_2O + 6C.$$

The activity of these bodies depends upon the strength of the affinity for water, sulphuric acid heading the list, and the least deliquescent substance at its foot. Another factor is, however, to be taken into consideration,—viz., the chemical reaction between the obtundent or active chemical agent and the protoplasm traversing the dentine. The effect of sulphuric acid has been noted. The powerful alkalies have the power of dissolving protoplasm or making a solution in which the character of the albumen is profoundly altered.

The metallic salts, zinc chloride, and silver nitrate enter into combination with the albumen, forming bodies indefinitely known as albuminates of the several metals; carbolic acid and allied substances have an analogous power,—in other words, they effect the coagulation of the albumen present. The extent of this coagulation may be seen in a series of experiments of Dr. James Truman (International Dental Journal, January, 1895). The results of this series of experiments are of direct practical interest and clinical application. As stated under the heading, agents which effect coagulation produce a chemical change in the contents of the dentinal tubuli, which means their destruction,—that is, the destruction of the terminals of the receiving apparatus.[1]

---

[1] I noted once in an essay or discussion—whose I cannot recall—a statement which implied that zinc chloride and perhaps other hygroscopic salts possessed the power of abstracting water from even the basis substance of dentine. The statement is without foundation. The zinc salt certainly possesses a strong

The parts to which these agents are applied become anæsthetic because of the necrosis induced. Viewing the reaction as a matter of chemistry, it is, of course, evident that the extent or depth of the coagulation will depend upon the amount of coagulant used and the length of time of application.

It is needless to enlarge here upon the general clinical experience that the use of the destructive agents is frequently necessary. As a group these agents comprise a class which finds clinical application only when milder means of obtaining sensitivity have failed; and, again, the agents which effect the least chemical alteration are employed before applying those of greater chemical activity. For example, carbolic acid or one of its derivatives, if found ineffective, would be succeeded by applications of sodium carbonate, and next by zinc chloride.

Another hygroscopic preparation should receive at least mention in this connection,—that is, the glycerole of tannin. Both ingredients of this prescription exert their specific effects in obtunding.

There is a point in the investigations and experiments of Dr. Truman which is of great significance,—*i.e.*, it has always been believed that silver nitrate was very superficial in its effects. The experiments as to its effects in coagulation have shown it to be one of the most penetrating of coagulants. This finding will necessarily modify our opinions as to the therapeutic use of this salt. I do not enter into the question of the diffusibility of coagulants, Dr. Kirk's experiments and evidence having shown that the first layer of coagulum formed by contact of albumen and a coagulant does not prevent the passage of the latter through the coagulum; that the question is one of chemical affinity more than of physical diffusion.

---

affinity for water where it exists as water, but that it can decompose the basis substance of dentine is, of course, not true. The same essay, if I recall the statements correctly, implied that zinc chloride possessed such an affinity for water that it continued its action until its affinity in this direction was unsatisfied,—*i.e.*, to an indefinite dilution. This, if generally believed, is a serious error. Such a belief implies that, if, say, one hundred grains of zinc chloride (fused) were added to a solution of albumen, say ten thousand grains, the fluid found after the disappearance of the solid salt would still contain the one hundred grains of chloride diluted by the water of the solution and of the albumen; that the coagulum which is formed would be formed in consequence of the abstraction of water by the salt. As a matter of fact, the amount of salt in solution would be found to be diminished by just the amount required to form the albuminate of zinc, so that if there be an excess of albumen the zinc will have disappeared from the solution and may be recovered from the coagulum.

The last class of agents used for the purpose under discussion are those which benumb or temporarily abolish the function of the receptive apparatus, these form the group of the obtundents proper. So far as clinical evidence is concerned, these agents act as specific obtundents without demonstrably altering the structure of the parts to which they are applied.

This sentence of course is to be accepted in its broad sense, for it is probable that some chemical reaction does occur between drugs and protoplasm which it is beyond the resources of experimental pharmacology to determine.

Nearly all of the agents of this group are included in the essential oils and the powerful alkaloids. Chloroform should be mentioned, however, as possessing the obtundent property.

The essential oils were probably among the first agents to find employment, if not for this specific purpose, they were at least applied for the relief of other dental pains. Those found to possess marked obtunding properties are the oils of cloves, cinnamon, gaultheria, thymol, myrtol, and several others. Of these, thymol appears to be the most effective. Their effectiveness, as a rule, increases in the degree that the pulp is approached.

Before passing to a discussion of the uses of the alkaloids in this connection, space should be accorded the mechanical means of preventing, not obtunding, pain,—that is, the rapid, light, and interrupted cutting of the dentine by means of perfectly true and perfectly sharp burs. The physical injury implied is marked by less irritation under this means than when dull or untrue burs are used.

The alkaloids which have been employed as obtundents are veratria, aconitia, atropia, and cocaine. While all of these bodies have been found to reduce the sensitivity of an exposed tooth-pulp, when applied locally to the dentine itself, the analgesic effect has not been sufficiently marked to term them obtundents so far as hypersensitive dentine is concerned.

The nearest approach to such an effect being noted in connection with cocaine; to secure the obtunding effect in favorable cases, the ordinary solutions, even saturated, have been found inefficient, the cocaine crystals are made into a thick paste with glycerin and applied to the dentine. Dr. F. D. Gardiner has, I believe, the records of success with this method.

The other alkaloids named have been used in weak solution, rarely in strong preparations, owing to the danger of toxic effects should such powerful agents get into the mouth and be swallowed. They are, therefore, in view of the risk involved, deemed dangerous agents for dental experimentation; although aconitia and veratria are powerful analgesics, even in small doses.

As these alkaloids are applied to act upon the terminals of the dentinal fibrillæ, when used in combination with the powerful coagulants, it is impossible to determine which agent has induced the analgesia.

Another agent which has been employed as an obtundent is fitly placed in a class by itself. I allude to arsenic and preparations which contain it. That this agent will most certainly destroy entirely the sensitivity of the dentinal fibrillæ is beyond a question, but there is good evidence that its application, even for short periods, is almost invariably followed by death of the pulp itself. Application of arsenic at a distance from the pulp is followed in some instances by throbbing pain, which would seem to indicate that a reflex hyperæmia had been induced; in other cases no sensation whatever is produced, and yet paralysis and degeneration of pulp-tissue, characteristic of the action of arsenic, are found to occur.

Dr. Henry C. Register has found that Fowler's solution, the liquor potassii arsenitis, has the power of promptly and certainly abolishing the sensitivity of the dentinal fibrillæ; it is presumed that, as with arsenous acid, the effect is necrotic.

Although the obtundent effect of arsenical preparations is undoubted, almost universal opinion debars their use, except in cases where the inevitable pulp devitalization is desired.

The next agent to be considered as an obtundent is the electric current itself. It is a well recognized fact in medical practice that the constant or galvanic current has the power of alleviating pain; it has been in general use in medicine for the treatment of various neuralgias, its minimum of success attending its application in neuralgia of the trigeminus. The interrupted current was used in the fifties to render painless the extraction of teeth, and successfully in a proportion of cases.

Dr. W. G. A. Bonwill states that in 1859 he procured a patent for an induction apparatus with a constant current, for the production of dentinal analgesia.

In the *Dental Cosmos*, 1875, Dr. J. Foster Flagg described a primary induction apparatus which he had used previously, and does use to the present day for the obtunding of the hypersensitivity of dentine. Experiments with this apparatus, which he terms the dental, have shown that it induces insensitivity of hyperæsthetic dentine in a very few minutes.

The latest development in this field, or, as it might be more accurately denominated, the readoption of electrical osmosis, has occupied the space it deserves in journalistic literature. To this literature the writer has nothing new to add except a belief in the benign influence of cocaine carried into the pulp by such means; however, in consideration of the fact that constant currents in themselves have been shown to exert an analgesic influence, it would be interesting if those who are experimenting with the cataphoric current were to try its effects without the addition of the cocaine.

An essay upon the present subject should naturally touch upon, if not discuss, the nature of the nerve terminals of the dental pulp. Whatever anatomical connection there may be, and I believe it to be a close one, between the odontoblasts and the plexus of nerve-fibres immediately underlying them, it is certainly true that, so far as physiological function is concerned, the odontoblastic processes behave like sensory nerve terminals. It is my expectation that with growing refinement of histological technology it will be found that the terminals of these nerves are in the odontoblasts themselves, I cannot see any other explanation of the phenomena observed in hyperæsthetic dentine.

